# Accuracy of Broiler Activity Index as Affected by Sampling Time Interval

**DOI:** 10.3390/ani10061102

**Published:** 2020-06-26

**Authors:** Xiao Yang, Yang Zhao, George T. Tabler

**Affiliations:** 1 Department of Agricultural and Biological Engineering, Mississippi State University, Mississippi, MS 39762, USA; xy123@msstate.edu; 2 Department of Poultry Science, Mississippi State University, Mississippi, MS 39762, USA; ttabler@poultry.msstate.edu

**Keywords:** broiler, activity index, time interval, age, image processing

## Abstract

**Simple Summary:**

Broiler activity index is a measure of bird movement through determining bird-representative pixel changes between consecutive images. Since the concept of activity index was coined, different sampling time intervals of consecutive images have been used to determine broiler activity. In this study, we found that accuracy of broiler activity decreased at longer sampling time intervals, with the 0.04-s interval yielding the most accurate activity index among all intervals investigated. In addition, broiler activity in the commercial house generally decreased as birds aged and varied at different monitoring locations. The research provides insights into image-sampling strategies for accurately determining broiler activity index, which may help to address growing public concerns on poultry welfare and health.

**Abstract:**

Different time intervals between consecutive images have been used to determine broiler activity index (AI). However, the accuracy of broiler AI as affected by sampling time interval remains to be explored. The objective of this study was to investigate the effect of the sampling time interval (0.04, 0.2, 1, 10, 60, and 300 s) on the accuracy of broiler AI at different bird ages (1–7 weeks), locations (feeder, drinker, and open areas) and times of day (06:00–07:00 h, 12:00–13:00 h, and 18:00–19:00 h). A ceiling-mounted camera was used to capture top-view videos for broiler AI calculations. The results show that the sampling time interval of 0.04 s yielded the highest broiler AI because more bird motion details were captured at this short time interval. The broiler AIs at longer time intervals were 1–99% of that determined at the 0.04-s interval. The broiler AI at 0.2-s interval showed an acceptable accuracy with 80% less computational resources. Broiler AI decreased as birds aged but increased after week 4 at the drinker area. Broiler AI was the highest at the open area for weeks 1–4 and at the feeder and drinker areas for weeks 5–7. It is concluded that the accuracy of broiler AI was significantly affected by sampling time intervals. Broiler AI in commercial housing showed both temporal and spatial variations.

## 1. Introduction

Broiler activity is considered a major indicator of animal physical and physiological conditions [1,2]. It was reported by Thorp and Duff [3] that exercising broilers a few times every day could benefit broilers’ leg skeletal conditions [4], thus reducing the incidence of lameness and improving bird walking ability [5]. More movement and activity by broilers may also help to reduce the prevalence of hock burns [6], footpad dermatitis [7], and breast burns [8] by reducing the contact with wet litter.

In order to quantify the animal activity, activity index (AI), a measure of movement intensity through image processing, was proposed by Bloemen et al. [9]. Activity index was defined as the percentage of pixels of moving animals to the total number of pixels within the image (including animals and background). In more recent research, the total number of pixels was replaced with total bird-representative pixels to compensate for variations in animal size at different ages [10,11,12]. Since the concept was coined, AI has been widely used to quantify the activities of broilers [13,14].

Broiler AI is calculated by determining changes in bird-representative pixels between consecutive images. Different from the optical flow statistics that Dawkin et al. [15] used to derive measures of broiler behaviors and gaits in commercial farm, the method of AI in this study only considers the amount of movement between consecutive images while the movement direction is not included. Using a short time interval between consecutive images may capture more movement details and yield better AI accuracy; however, a time interval that is unnecessarily short (e.g., yielding too many consecutive images for only trivial bird movements) cannot further improve AI accuracy and may increase image processing time. Longer time intervals, on the other hand, may miss identifying birds’ movements and compromise AI accuracy. Therefore, a proper time interval is important for ensuring the accuracy of AI while improving processing efficiency and saving computational resources. Different time intervals have been used to determine broiler AI in previous research. For instance, Neves et al. [14] analyzed the images at 60 s intervals to determine bird activity as affected by feeder types. Bloemen et al. [9] sampled images with a time interval of 5 s to investigate the effect of the thermal environment on broiler activity. Aydin et al. [10] used a time interval of 0.2 s to measure the activity of broilers with different gait scores. However, proper time intervals remain to be explored.

Selection of a proper time interval should be based on the birds’ movement intensity, which could be affected by many factors, such as bird age, location, and time of day. Bird movement intensity may vary with bird age due to changes in their physical conditions, like body weight and walking ability [11,16]. Broilers do not always spread out evenly on the floor areas in commercial houses, resulting in different degrees of crowdedness that may affect bird movements. For example, Arnould et al. [17] found that areas near feeders and drinkers were more crowded because broilers tended to stay and rest near the sources of feed and water. Furthermore, bird movement intensity varies within a day. Previous research showed that birds are more active during the first hour after light ON and before light OFF [18]. However, the effects of the above-mentioned temporal and spatial variations in broiler movement intensity on broiler AI have not been investigated.

The objective of this study was to investigate the effect of sampling time interval (0.04, 0.2, 1, 10, 60, and 300 s) on the accuracy of broiler AI. The effects of bird age (1–7 weeks), location (feeder, drinker, and open area) and time of day (06:00–07:00 h, 12:00–13:00 h and 18:00–19:00 h) on broiler AI were also examined.

## 2. Materials and Methods 

### 2.1. Housing, Animals and Management

The study was conducted in a commercial broiler house located at Mississippi State University during 12/2019–1/2020. The house measured 120 × 13 × 3 m (L × W × H) with a capacity of 16,120 Ross 708 straight run broilers and a production cycle of 8 weeks. All chicks were purchased from a commercial poultry hatchery in Mississippi. Both tray and tube feeders were used in weeks 1 and 2 of bird age, then tray feeders were removed from week 3. Flock management and diets followed the typical procedures in the industry. The lighting schedule was set to 24L:0D from 1 d to 7 d, 20L:4D from 8 d to 56 d. The light intensity was set to 54 lux from 1d to 13 d, then gradually dimmed to 3 lux by 20 d and kept at 3 lux till 56 d. Lights were turned on at 05:00 h and turned off at 01:00 h of the next day.

### 2.2. Camera System

**Figure 1:**
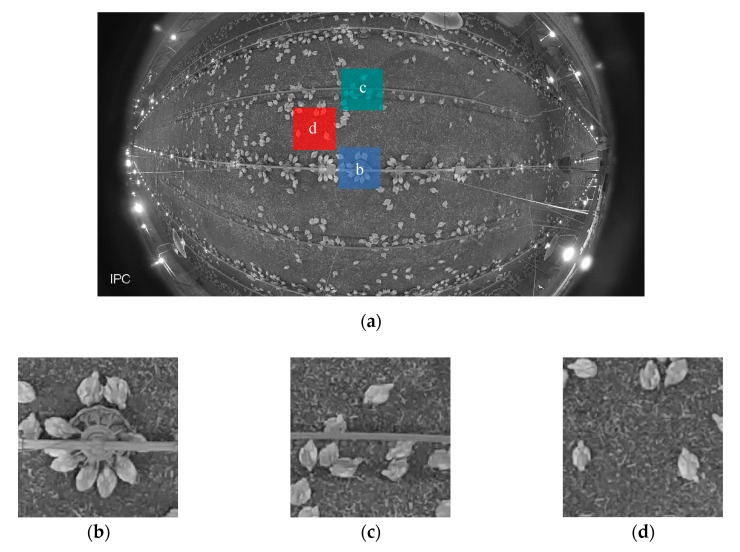
Example images of (**a**) original image (**b**) feeder (**c**) drinker and (**d**) open area.

A fisheye IP camera (Dahua, IPC-EW4431-ASW, Dahua Technology USA Inc., Irvine, CA, USA) was installed on the ceiling (height = 3 m), located in the middle of the house. The frame rate of the camera was 25 frames per second. Three one-hour video clips (06:00 to 07:00 h, 12:00 to 13:00 h and 18:00 to 19:00 h) were recorded on Wednesday every week. The video clips were converted into images with all frames extracted (time interval of 0.04 s between consecutive images) or partial frames at time intervals of 0.2, 1, 10, 60 and 300 s. Images were firstly corrected for distortions using Python (Python 3.7.1, Python Software Foundation, Beaverton, OR, USA). Afterwards, three specific areas (200 × 200 pixels) located at the feeder, drinker and open area (Figure 1) were cropped out of the images and fed to MATLAB (2018b, The MathWorks, Inc. Natick, MA, USA) for image processing. The dimension of cropped area was equivalent to the actual area of 0.71 × 0.71 m. The images were used to calculate AI at the drinker and open areas in weeks 1–7, and at feeding area in weeks 3–7 when only tube feeders were provided. 

### 2.3. Activity Index

The value *I*(x, y) represents the intensity of the pixel at coordinates (x, y) in that image. The difference in intensity between the current image *I*(x, y, t) and the previous image *I*(x, y, t − 1), was calculated by subtraction of the two consecutive images. A resulting image of *I_a_*(x, y, t), in binary form, was then generated according to results of the subtraction (Equation (1)).

(1) $$I_{a}\left(x,y,t\right)=\{\begin{array}{l} 1,\;\;if\;I\left(x,y,t\right)-I\left(x,y,t-1\right)>\tau \\ 0,\;\;otherwise \end{array}$$

The threshold (*τ*) was set to 15% of the maximal intensity of each video clip by observing the “empty” background of first 20 frames for each clip to avoid erroneous results due to noise, e.g., electrical noise in the coaxial cabling and image acquisition circuits, and lighting variations. 

Total number of non-zero pixels was calculated as the variation between the two consecutive images due to the activity of broilers. To compensate for the size and number of the birds, the activity index AI(t) was calculated as the fraction of the number of non-zero pixels in the resulting binary image *I_a_*(x, y, t) with respect to the number of broiler-representative pixels S(t − 1) in the previous image *I*(x, y, t − 1) (Equation (2)).

(2) $$AI\left(t\right)=\frac{\sum I_{a}\left(x,y,t\right)}{S\left(t-1\right)}$$

### 2.4. Data Preparation and Statistic Analysis

The effects of sampling time interval, bird age, sampling location, and time of the day, as well as the major two-way interactions, on broiler AI was analyzed using the PROC GLM (generalized linear model) procedure in SAS 10.9 (SAS Institute., Cary, NC, USA). A significant difference in multiple comparisons of group means was defined as *p* < 0.05. The levels for time intervals were 0.04, 0.2, 1, 10, 60 and 300 s. The levels of age were 1–7 weeks in drinker and open areas and 3–7 weeks in the feeder area. The levels of sampling location factor consisted of feeder, drinker and open areas. The levels of sampling time within a day were 06:00–07:00 h, 12:00–13:00 h and 18:00–19:00 h. In order to compare the difference of AI among different time intervals, the cumulative AI of every 300 s was calculated by simply adding the AIs within a 300-s duration. Some of the video clips were not strictly an hour long (56 to 59 min); therefore, 11 total samples were obtained from each video clip. For the effects of bird age, location and time of day, only data with time intervals of 0.04 s (full frames) were used for analysis.

## 3. Results 

### 3.1. Time Interval

Table 1 shows the average broiler AI with different time intervals at different locations. A time interval of 0.04 s yielded the highest AI. With an increase in time interval, the broiler AI deceased from 100% (0.2 s) to 2% (300 s) of the AI determined with a time interval of 0.04 s (*p* < 0.0001 for all). No differences in AI were observed between the ratio of 0.04 s and 0.2 s at the feeder and drinker areas. However, a lower ratio of AI at the 0.2-s interval was found at the feeder area compared with the 0.04-s interval ((*p* < 0.0001). Lower broiler AIs were observed at the 1-s time interval than at the 0.2-s interval at the feeder (*p* < 0.0001) and drinker (*p* = 0.0267) areas. At the open area, the broiler AI with a time interval of 1 s was lower than 0.04 s (*p* = 0.0007); however, no significant difference was observed between 0.2 s and 1 s. 

### 3.2. Age and Location

Table 2 shows the weekly average broiler AI at different locations. At the feeder area, the broiler AI in week 3 was higher than that in weeks 4 and 7 (*p* < 0.0001). No significant differences were observed in weeks 4, 6 and 7. At the age of week 5, the AI was higher than in weeks 4 (*p* = 0.0024) and 6 (*p* = 0.0218), however, not different from week 7. Broiler AI at the open area gradually decreased from week 1 to week 5, and no differences were found in weeks 4, 5, 6 and 7. No difference in broiler AI at the open area was observed between weeks 2 and 3. The AIs in weeks 4–7 were lower than those in weeks 1–3 (*p* < 0.05). For the effects of sampling locations, the broiler AIs at the open area were higher than at the drinker area in weeks 1 and 2 (*p* = 0.0098 and *p* = 0.0011, respectively). In weeks 3 and 4, the highest broiler AI was observed at the open area and the lowest AIs at the drinker area (*p* < 0.0001). In weeks 6 and 7, the broiler AIs at the feeder (*p* = 0.0011 and *p* = 0.0134, respectively) and drinker (*p* = 0.0004 and *p* < 0.0001, respectively) areas were higher than at the open area. Generally, broiler AI decreased as broilers aged up in all locations in this study. 

### 3.3. Time of Day

Table 3 shows the weekly average broiler AI within three time periods at three locations. At the feeder area, differences among time periods were observed at 5 and 6 weeks of bird age. At the drinker area, higher broiler AIs were observed at either 12:00 h or 18:00 h during 1–7 weeks of bird age. The lowest broiler AIs were observed at 06:00 h, except in week 3 at the drinker area. At the open area, differences were found in weeks 4 and 6, with the highest AIs being identified at 12:00 h and the lowest at 06:00 h (*p* = 0.0007 and *p* = 0.0026, respectively). 

### 3.4. Selection for Proper Time Interval

**Figure 2:**
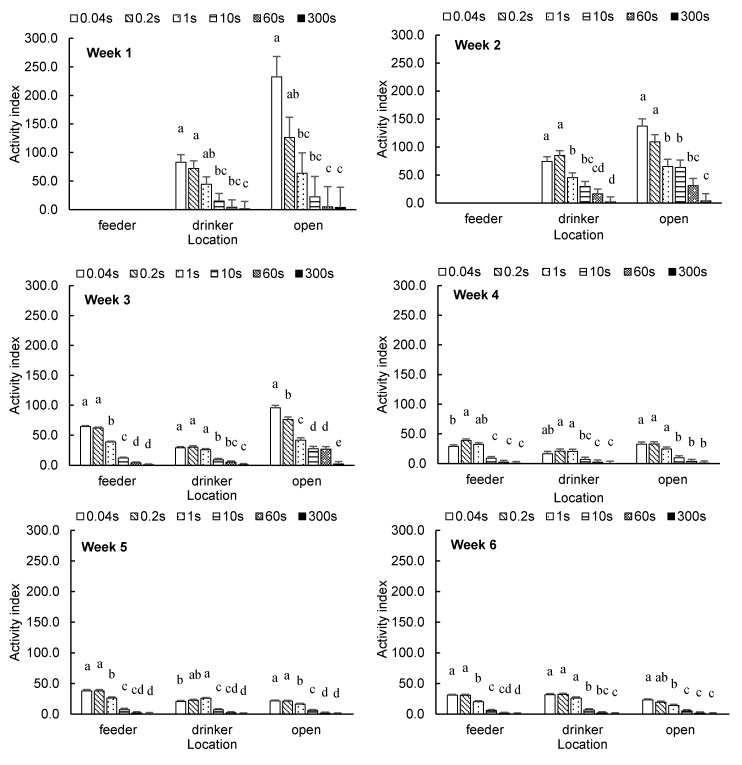

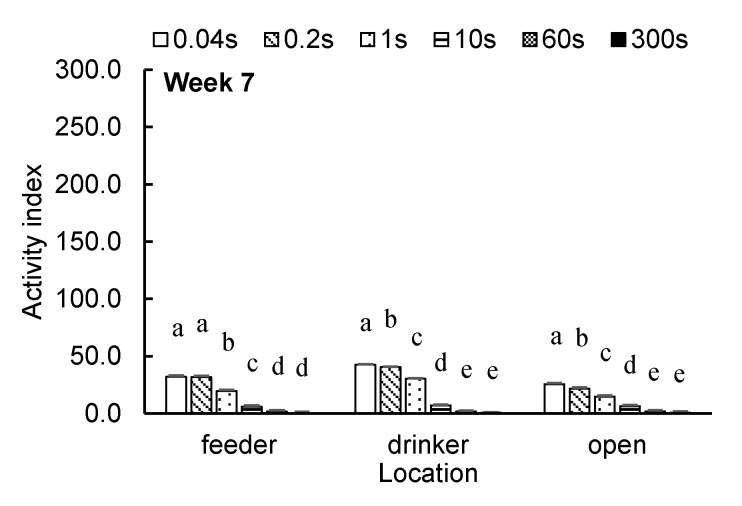
Weekly average broiler activity index (least square means ± SEM) at feeder, drinker and open areas with different time intervals. ^a,b,c,d,e^ Means with different superscripts within the same category are different (*p* < 0.05).

Figure 2 shows the weekly average broiler AI at the feeder, drinker and open areas with different time intervals. At the feeder area, no difference in broiler AI was observed between the time intervals of 0.04 s and 0.2 s in week 3 or weeks 5–7. In week 4, the AI with the time interval of 0.2 s was higher than with 0.04 s (*p* = 0.0320). At the drinker area, the difference in broiler AI in weeks 1–4 and week 6 was not significant between 0.04-s and 0.2-s intervals. In week 5, the AI with the time interval of 1 s was higher than that with 0.04 s (*p* = 0.0436). At week 7, a time interval of 0.2 s yielded a lower AI than 0.04 s (*p* = 0.0068). At the open area, no differences in broiler AI between time intervals of 0.04 and 0.2 s were found in weeks 1–2 or weeks 4–6. In weeks 3 and 7, the AI with a time interval of 0.2 s was higher than for 0.04 s (*p* = 0.0056 and *p* = 0.0159). 

## 4. Discussion

Different sampling time intervals, from seconds to minutes, have been adopted to calculate the AI of livestock and poultry [10,12,19,20]. However, how the time intervals affect the accuracy of animal AI remains to be understood. In this study, we found that AI decreased at longer sampling time intervals (Table 1). This is because some transient behaviors, such as turning, bobbling, preening and shaking, can be miss-identified as the sampling time interval increases. Any time interval that lasts longer than the time of the transient behavior may fail to capture the information. Our results also show that a 0.2-s interval can be considered as an alternative to 0.04 s in broiler AI determination, because it delivered comparably high AI while reducing the image processing workload by 80%. Selection of proper sampling time intervals for accurate AI measurement of certain animal species should consider the velocities of both continuous movements (e.g., walking and running) and discrete movements (e.g., pecking, touching, smelling, dashing, etc.). A sampling time interval of 0.03 s has been used for cows [19,21], 0.04 s for pigs [20,22], and 0.2 s and 0.3 s for poultry [10,12,23].

Broiler AIs generally decreased as the birds got older (Table 2). This result is consistent with those previously reported by Alvino et al. [24] and Bizeray et al. [25], who also found older broilers were less active while gaining body weight [26]. Increases in AIs at the drinker and feeder areas were noticed after week 4 of bird age (Table 2). This is possibly because of birds’ increasing demand for feed and water [27] as birds get older and heavier, promoting more traffic around drinkers and feeders. 

Our results show obvious spatial variations in broiler AI, which can be explained by the different movement intensities of predominant behaviors in the locations of concern (i.e., feeder, drinker, and open areas). Feeding and drinking behaviors at feeder and drinker areas [28,29] do not involve more intensive body movements compared to walking, chasing, and playing behaviors (sparring, frolicking and food-running) [30] at open litter areas. Therefore, the broiler AIs in feeder and drinker areas were lower than that in open areas in weeks 1–4 (Table 2). As broilers got older and heavier, they became less active and tended to use the open area as a place for resting, which involves less body movement than feeding and drinking. As such, we found a lower broiler AI in open areas, as compared to feeder and drinker areas, in weeks 5–7. 

Understanding the broiler AI at the feeder area may also help to clarify the birds’ health status. For instance, Weeks et al. [31] compared the feeding patterns of broilers with different gait scores and found those birds with substantially impaired walking ability halved the number of feeding bouts but doubled the feeding time per visit to reach the same amount of feed intake as healthy birds. It should be noted that the AI in our study quantifies the bird activity within a 0.71 × 0.71 m area (including both the feeder/drinker and surrounding areas); thus, it involves not only the feeding and drinking behaviors but also the traffic around feeders and drinker lines. This is different from other measures of feed and water resource usage by broilers, such as feed intake [32] time budget of broiler feeding and drinking behaviors [33,34], and feeding and drinking frequency (times of feeder or drinker visiting within an hour) [35,36]. 

There were also temporal variations in broiler activity within a day (Table 3). The possible explanation for higher broiler AI at the feeder area in the early morning or afternoon could be the broiler diurnal rhythms. Li et al. [37] explored the effect of light intensity and spectrum on broiler (35–40 d) feeding behaviors with a schedule of 16L:8D and found the peak feeding occurred at 2–3 h after light ON and before light OFF. The high level of feeding behavior in the early morning could be a feed compensation after the dark period without any feed intake [38]. More feeding behavior at the end of a day was probably because the birds can anticipate the darkness [39] and spontaneously increase the feed intake before the light is turned OFF [28]. It should be pointed out that the broiler AI at the feeder area during 06:00–07:00 h did cover the early light ON period but was not consistently higher than that of the midday period. The discrepancy is possibly because of the difference in bird age or lighting schedule [40]. In addition, the movement of birds around the feeder, which was equivalent to the movement of birds at the open area, could be another reason for higher AI during 12:00–13:00 h at the feeder area. There was no clear temporal pattern in AI at the drinker area based on our results. Schwean-Lardner et al. [41] studied the effect of daytime length on broiler (27 d and 42 d) behavioral patterns and found a similar rhythm of drinking behavior as of feeding. In another study that explored the effect of feeding and lighting programs on broiler feeding and drinking patterns indicated that the drinking patterns of broilers were largely influenced by lighting programs but independent of feeding behaviors [40]. One explanation for the discrepancy could be the lighting schedule. Another reason could be the movement of birds near the drinker line within the concerned area. As for open area, higher broiler AI was typically found at middle of the day in our study, which is consistent with the results reported by Sherlock et al. [42].

It worth noting that the AI is a measure of bird movements in two dimensions on a horizontal plane. Bird movements in the vertical direction for behaviors such as standing up, lying down, and pecking the feeder and drinker are not included in the AI calculation, but may have important implications for poultry welfare, health, and production efficiency. To account for the birds’ vertical motion, cameras with a depth sensor or body-mounted sensors that produce 3D motion measurements may be considered [12]. 

## 5. Conclusions

In this study, the effects of sampling time interval between consecutive images on the accuracy of broiler AI at different bird ages, sampling locations and times of day were investigated using image processing. We conclude that a sampling time interval of 0.04 s yielded the best broiler AI results. The AI results for a 0.2-s time interval were acceptable but required significantly less computational resource usage. At different ages, broiler AIs at the feeder and open area generally decreased from week 1 to week 7. However, an increase at the drinker area was observed after week 4. For the effects of location, higher AIs during weeks 1–4 occurred at the open areas, and this switched to feeder and drinker areas during weeks 5–7. Clear diurnal behavioral rhythms were also found at feeder and open areas. In summary, broiler AIs in commercial housing showed both temporal and spatial variations. These findings provide important insights into accurate broiler activity measurement for broiler welfare, health, and production evaluation.

## Figures and Tables

**Table 1:** Average broiler activity index (AI) with different time intervals at different locations.

Time Interval (s)	Location					
	Feeder		Drinker		Open Area	
	AI	Ratio (%)	AI	Ratio (%)	AI	Ratio (%)
0.04	38.8 ^a^	100	42.5 ^a^	100	81.2 ^a^	100
0.2	38.5 ^a^	100	41.7 ^a^	100	58.0 ^ab^	84
1	27.3 ^b^	74	31.1 ^b^	92	34.3 ^bc^	56
10	8.0 ^c^	21	11.7 ^c^	32	20.1 ^cd^	27
60	2.3 ^cd^	6	4.9 ^cd^	12	10.2 ^cd^	13
300	0.6 ^d^	1	0.9 ^d^	2	1.7 ^d^	2
SEM	3.1	2	5.4	4	13.4	3

Ratio: AI calculated at a time interval relative to that at the 0.04-s time interval. SEM: pooled standard error mean for the main effects of location. ^a,b,c,d^ Means in the same column with different superscripts are different (*p* < 0.05). Five weeks (week 3–7) of data in the feeder area and seven weeks (week 1–7) of data in the drinker and open areas are summarized.

**Table 2:** Weekly average broiler activity index (AI) at different locations.

Bird Age (Week)	Location			
	Feeder	Drinker	Open	SEM ^1^
1	--	83.2 ^Ba^	232.5 ^Aa^	39.7
2	--	74.1 ^Ba^	137.2 ^Ab^	13.0
3	64.5 ^Ba^	28.9 ^Cbc^	95.8 ^Ab^	7.2
4	28.6 ^Ac^	16.5 ^Bc^	32.6 ^Ac^	1.9
5	37.8 ^Ab^	20.7 ^Bc^	21.6 ^Bc^	1.7
6	30.9 ^Ac^	31.5 ^Abc^	23.1 ^Bc^	1.6
7	31.9 ^Bbc^	42.4 ^Ab^	25.5 ^Cc^	1.8
SEM ^2^	2.1	6.1	14.8	--

Data with a time interval of 0.04 s were included. ^1^ SEM: Pooled standard error mean for age effect. ^2^ SEM: Pooled standard error mean for location effect. ^A,B,C^ Means in the same row with different superscripts are different (*p* < 0.05). ^a,b,c^ Means in the same column with different superscripts are different (*p* < 0.05).

**Table 3:** Weekly average broiler activity index (AI) within three time periods (06:00–07:00 h, 12:00–13:00 h, and 18:00–19:00 h) at three locations (feeder, drinker, and open area).

Bird Age (Week)	Location											
	Feeder				Drinker				Open			
	06:00	12:00	18:00	SEM	06:00	12:00	18:00	SEM	06:00	12:00	18:00	SEM
1	--	--	--	--	43.7 ^B^	136.3 ^A^	69.5 ^B^	21.0	179.0	384.9	133.7	91.0
2	--	--	--	--	46.0 ^B^	97.6 ^A^	78.7 ^AB^	11.4	91.7	159.0	160.7	28.3
3	60.2	64.0	69.2	6.4	31.6 ^A^	35.9 ^A^	19.1 ^B^	3.1	98.8	85.9	102.6	20.8
4	24.3	30.5	31.1	2.6	6.1 ^C^	19.6 ^B^	23.8 ^A^	1.3	22.5 ^B^	42.3 ^A^	32.9 ^AB^	3.7
5	43.4 ^A^	31.7 ^B^	38.3 ^AB^	2.3	18.7	19.1	24.2	3.1	18.5	26.3	20.0	3.2
6	28.8 ^B^	28.2 ^B^	35.7 ^A^	2.2	25.1 ^B^	31.8 ^AB^	37.8 ^A^	2.9	17.3 ^B^	29.1 ^A^	22.8 ^AB^	2.5
7	28.9	32.2	34.6	2.8	41.3	44.2	41.7	3.2	28.7	23.6	24.1	3.5

SEM: Pooled standard error mean for main effects of time periods at each location. ^A,B,C^ Means in the same row with different superscripts under the same location category are different (*p* < 0.05). Data with a time interval of 0.04 s were used.
